# A transcriptome-guided genetic engineering strategy to balance cell growth with astaxanthin production in *Phaffia rhodozyma*

**DOI:** 10.3389/fmicb.2025.1636554

**Published:** 2025-09-18

**Authors:** Jianping Jia, Chenxi Ye, Sainan Jin, Qingqing Li, Zhengyi Pan, Wen Wen, Guoliang Bao

**Affiliations:** School of Pharmacy, School of Food Science and Engineering, Hangzhou Medical College, Hangzhou, China

**Keywords:** *Phaffia rhodozyma*, astaxanthin, nitrogen-deficiency, oxidative stress, non-homologous end-joining, ferric reductase

## Abstract

Astaxanthin is a strong antioxidant and is widely applied in food industry. The yeast *Phaffia rhodozyma* is an ideal microbial astaxanthin resource. However, the nitrogen-deficiency stress, which is beneficial for astaxanthin synthesis, often impairs cell growth, leading to low productivity. In this study, an imbalance between cellular growth and astaxanthin synthesis in *P. rhodozyma* under nitrogen-deficient (H) and nitrogen-sufficient (L) conditions was identified. A comparative RNA-seq transcriptome analysis between the H and L groups revealed well-discriminated patterns. The differentially expressed genes (DEGs) indicated that the regulation of nitrogen deficiency does not occur directly in the astaxanthin biosynthesis pathway but rather operates at the global cellular level, involving processes such as central and energy metabolism, antioxidative stress responses, signal transduction, competitive metabolic pathways, and material transportation. Based on these findings, a regulatory mechanism is proposed, which involves cellular sensing of nitrogen sources in the medium, alterations in signaling pathways that direct effectors, and the regulation of multiple downstream target genes through post-translational modifications, protein interactions, gene transcription, and the protein and metabolite levels. Six DEGs were overexpressed in the wild strain (WT) of *Phaffia rhodozyma*, and the mutants M2 and M6, expressing the NHEJ gene for DNA repair and the ferric reductase gene, showed higher biomass and astaxanthin content compared with the WT strain under nitrogen-deficient conditions. However, the remaining mutants exhibited unchanged or even reduced biomass and astaxanthin productivity. Subsequently, a co-expression mutant (M7) carrying the two DEGs was constructed. This mutant exhibited further increases in both biomass and astaxanthin content, with 61.5 and 133.3% higher yields than the WT strain, respectively, and a 265.8% increase in final astaxanthin production.

## Introduction

Astaxanthin is a reddish-orange ketocarotenoid with the molecular formula C_40_H_52_O_4_ and the chemical name 3,3′-dihydroxy-4,4′-diketo-*β*,β′-carotene. Its long conjugated polyolefin chain can burst single-linear oxygen, scavenge free radicals, improve cell activity, and protect human liposomes, thus contributing to improved immunity and anti-aging effects. In addition, astaxanthin is the most powerful antioxidant, with 6,000 times more antioxidant power than vitamin C. Moreover, it can prevent most of the oxidative stress and related inflammation, including hypertension, cancer, obesity, cardiovascular disease, inflammatory disease, bone disease, and skin disease, and thus can be used in functional foods, medicines, aquaculture, and cosmetic industries (Mussagy et al., 2023). The global market value of astaxanthin, the second largest carotenoid, is expected to grow to nearly USD$3.4 billion by 2027 (Park et al., 2018). Currently, astaxanthin is mainly produced by natural extraction, chemical synthesis, and microbial fermentation. Natural extraction is used to extract astaxanthin from crustacean waste, such as lobster and crab, but the yield is very low. Meanwhile, foods like shrimp, crab, algae, fish, and shellfish also contain astaxanthin, but the content is not enough to meet human needs (Li et al., 2023). Although more than 90% of the astaxanthin on the market today is chemically synthesized, chemical synthesis is an environmentally unfriendly process, and its product is a mixture of astaxanthin with various configurations and by-products; thus, it is not approved for human use (Li et al., 2023). Microbial production of astaxanthin has the advantages of a clear configuration, environmental friendliness, and few byproducts; thus, some microorganisms, especially microalgae, such as *Haematococcus pluvialis,* yeast, such as the wild strain *Phaffia rhodozyma*, and the cell chassis, *Saccharomyces cerevisiae*, *Yarrowia lipolytica*, and *Komagataella phaffii*, are excellent astaxanthin producers.

*P. rhodozyma* (also known as *Xanthophyllomyces dendrorhous*) can produce astaxanthin in free form with the 3R, 3R’ isomer, which is easier to be purified and digested by humans. Moreover, astaxanthin is the predominant carotenoid contributing to 90% of the total carotenoids in *P. rhodozyma*. In addition, *P. rhodozyma* has the advantage of rapid self-propagation and cultivation in simple conditions and media and is not influenced by weather and climate. Therefore, *P. rhodozyma* has advantages over other microorganisms for astaxanthin production (Li et al., 2025). The astaxanthin synthesis pathway in *P. rhodozyma* has been completely elucidated. Initially, acetyl-CoA is converted into geranylgeranyl diphosphate (GGPP) via the 8-step reaction in the mevalonate (MVA) pathway. Subsequently, GGPP is then converted into *β*-carotene through a three-step reaction. Finally, β-carotene undergoes an oxidation reaction catalyzed by astaxanthin synthase (crtS) to yield astaxanthin (Kizer et al., 2008). Furthermore, the acetyl-CoA, NADPH, ATP, and Mg^2+^ play a pivotal role in facilitating astaxanthin synthesis in *P. rhodozyma* (Basiony et al., 2022). Nevertheless, the relatively modest astaxanthin productivity exhibited by *P. rhodozyma* has presented a challenge to the advancement of the biosynthetic astaxanthin industry. Therefore, it becomes particularly important for the study of the regulation of astaxanthin synthesis in *P. rhodozyma* and its mechanism, as well as the genetic modification to construct yeast strains with high astaxanthin production (Jia et al., 2023, 2024).

Cultivation conditions, such as the composition of nutritional media, are the most elementary and important factors for astaxanthin synthesis in *P. rhodozyma*. A number of carbon sources, such as common glucose, xylose, and arabinose; cheap materials, such as mesquite pods extract, barley straw, and sugarcane bagasse; a number of nitrogen sources, comprising organic yeast extract, malt extract, and peptone; and inorganic compounds, such as NH_4_Cl, (NH_4_)_2_SO_4_, and NH_4_NO_3_, were used in different media to produce astaxanthin in *P. rhodozyma* (Mussagy et al., 2023). However, for microorganisms, nitrogen deficiency or high carbon/nitrogen (C/N) ratio stress conditions that promote efficient astaxanthin synthesis while often affecting cell growth, and for the intracellular product astaxanthin, this imbalance becomes a bottleneck limiting astaxanthin production. To break through this bottleneck, a two-stage fermentation strategy, with the first stage being nitrogen-sufficient for cell growth and the second stage being nitrogen-deficient for astaxanthin synthesis, was applied in microalgae *H. pluvialis* for astaxanthin production. A further transcriptomic analysis showed that the nitrogen-deficient stress condition induced the differentially expressed genes (DEGs) associated with photosynthesis, astaxanthin biosynthesis, nitrogen transport and assimilation, and proteasome pathway, while it inhibited the DEGs related to chloroplasts and nonessential proteins, indicating that nitrogen deficiency can regulate astaxanthin synthesis in *H. pluvialis* through multiple dimensions, such as photosynthesis, astaxanthin biosynthesis, nitrogen metabolism, and protein degradation (Huang et al., 2019). For an engineered *Yarrowia lipolytica* strain ST7403, high-oleic safflower spent oil was used as the sole carbon source, and a high C/N ratio medium containing up to 70% safflower oil was applied for up to 167 mg/L astaxanthin (Li et al., 2020). Unlike *H. pluvialis* and *Y. lipolytica*, C/N in the astaxanthin-synthesis medium for *P. rhodozyma* is not emphasized; however, C/N ratios in various media for *P. rhodozyma* are as high as those for *H. pluvialis* and *Y. lipolytica*, confirming that nitrogen deficiency or high C/N ratio medium is also suitable for astaxanthin synthesis in *P. rhodozyma* (Mussagy et al., 2023). Moreover, the molecular mechanism of nitrogen deficiency to enhance astaxanthin in *P. rhodozyma* is still unclear.

Genetic modification of the microbial strain is the other significant strategy for enforcing astaxanthin biosynthesis. As early as the 1990s, the genetic transformation system has been successfully established in *P. rhodozyma* (Adrio and Veiga, 1995). However, currently, strategies to improve astaxanthin yield in *P. rhodozyma* mainly depend on optimization of the fermentation conditions and random mutagenesis (Li et al., 2025). Genetic modifications of *P. rhodozyma* to increase astaxanthin production have also been reported; however, the targets of genetic modification are mainly within the astaxanthin biosynthesis pathway (Hara et al., 2014; Breitenbach et al., 2019; Li et al., 2025). Since understanding of the global regulatory mechanisms governing astaxanthin synthesis in *P. rhodozyma* remains in the early stage and a more comprehensive strategy for global regulatory modifications aimed at augmenting astaxanthin production, the genetic modification targets outside the astaxanthin biosynthesis pathway is very rare. In our previous study, mechanisms of exogenous salicylic acid (SA) and melatonin (MT) promoting astaxanthin synthesis at the global transcriptome level were illuminated in *P. rhodozyma*, and a large number of DEGs were identified. Then, the novel polyamine transporter gene *PT* and zinc finger transcription factor gene *ZFTF* were selected from DEGs to be overexpressed in *P. rhodozyma*. Thus, synergistic chemical treatment and genetic modification strategies were applied to enhance astaxanthin productivity without the expense of cell growth (Jia et al., 2023, 2024).

In this study, the effects of the C/N ratio in the medium, under both the nitrogen-deficient and -sufficient conditions, on biomass, astaxanthin content, and yield in *P. rhodozyma* were analyzed. Then, the transcriptomic mechanism of high C/N ratio or nitrogen deficiency enhancing astaxanthin productivity while inhibiting cell growth in *P. rhodozyma* is illuminated, and the DEGs induced or inhibited by the nitrogen deficiency condition are identified. Genes represented in groups of DEGs were overexpressed individually or synergistically in *P. rhodozyma* to construct mutants with high astaxanthin productivity. In this study, we aim to illuminate the global mechanism of the nitrogen deficiency stress condition enhancing astaxanthin synthesis and inhibiting cell growth and present stress treatment and genetic modification strategies to balance cellular growth and astaxanthin biosynthesis in *P. rhodozyma* for efficient astaxanthin production.

## Materials and methods

### Reagents

Astaxanthin was of HPLC grade, while all other reagents met at least analytical grade standards. All reagents were sourced from Shanghai Aladdin Biochemical Technology Co., Ltd. (Shanghai, China).

### Microbial strain, medium compositions, and culture conditions

The *P. rhodozyma* strain AS2.1557 was obtained from the China General Microbiological Culture Collection Center (Beijing, China). For seed culture, the strain was cultivated on a YPD medium containing 20 g/L glucose, 10 g/L yeast extract, and 20 g/L peptone. For fermentation, the basal medium composed of 5 g/L tryptone, 3 g/L yeast extract, 10 g/L malt extract, and 50 g/L glucose and is considered to have a normal C/N ratio of ~7.5 (the N medium), while the other fermentation media were prepared with glucose and malt extract fixed, and tryptone and yeast extract were adjusted into 1 g/L, 0.8 g/L, and 8 g/L, 4 g/L, respectively, yielding high C/N ratio (~33, the H medium) and low C/N ratio (~5, the L medium). The seed culture under the same conditions was transferred into the fermentative H, N, and L medium and maintained at 22°C and 300 rpm for 96 h, respectively. The cell from the three media was presented by the H, N, and L groups. Positive *P. rhodozyma* transformants were selected using YM medium plates composed of 5 g/L tryptone, 3 g/L yeast extract, 3 g/L malt extract, and 10 g/L glucose and supplemented with 50 mg/L G418.

### Cell growth assessment

The cell growth of *P. rhodozyma* AS2.1557 was quantified as dry cell weight (DCW). An aliquot of 50 mL of cell culture was centrifuged at 4 °C and 7,000 *g* for 5 min and then washed twice with distilled water. The cell pellets were subsequently lyophilized at approximately −50 °C for approximately 48 h, or until a constant weight was achieved (Yu et al., 2019).

### Astaxanthin extraction and analysis

A 3-mL volume of dimethyl sulfoxide (DMSO) was preheated to 60°C and thoroughly mixed with *P. rhodozyma* cells. The mixture was incubated at 50°C for 5 min, after which 3 mL of anhydrous ethanol was added, and the mixture was incubated for an additional 20 min. The sample was then subjected to extraction using ultrasonication for 10 min, followed by centrifugation at 8,000 rpm and 4°C for 10 min. This extraction process was repeated in several cycles until the cell pellet turned white. The supernatants from each cycle were collected and diluted to a final volume of 20 mL. Astaxanthin was analyzed using an Essentia LC-16 HPLC system equipped with a Hypersil BDS C18 column (Shimadzu, Japan). The detection wavelength for astaxanthin was 478 nm, with an injection volume of 10 μL, a column temperature of 25 °C, and a flow rate of 1 mL/min. The mobile phase consisted of methanol and acetonitrile in a 9:1 volume ratio (Pan et al., 2020).

### RNA extraction and library construction

Total RNA was extracted from each sample using TRIzol Reagent (Invitrogen, USA). The quantity and purity of the RNA were assessed with an Agilent 2,100 Bioanalyzer (Agilent Technologies, USA), a NanoDrop spectrophotometer (Thermo Fisher Scientific Inc., USA), and 1% agarose gel electrophoresis. The integrity of the RNA was evaluated using the Bioanalyzer 2,100, ensuring an RNA Integrity Number (RIN) greater than 7.0, which was further confirmed by electrophoresis on a denaturing agarose gel (Yu et al., 2019). Poly(A) RNA was purified using Dynabeads Oligo(dT)25–61,005 (Thermo Fisher, USA) through two rounds of purification. This purified RNA served as the template for first-strand cDNA synthesis with ProtoScript II Reverse Transcriptase (Invitrogen, USA). The second strand of cDNA was then synthesized using the Second Strand Synthesis Enzyme Mix, which included dACG-TP/dUTP, *E. coli* DNA polymerase I (NEB, USA), RNase H (NEB, USA), and dUTP Solution (Thermo Fisher, USA). An A-tail was added to both strands, followed by ligation with an adaptor containing a T-tail. The adaptor-ligated DNA underwent size selection using AMPureXP beads. After treatment with Uracil-Specific Excision Reagent (USER) enzyme (NEB, USA) to excise the U-labeled second-strand DNAs, the ligated products were amplified by PCR for 10 cycles. Both primers used in the PCR reaction were designed to bind to the flow cell for bridge PCR. The resulting PCR products were purified using AxyPrep Mag PCR Clean-up (Axygen, USA), validated using an Agilent 2,100 Bioanalyzer (Agilent Technologies, USA), and quantified with a Qubit 2.0 Fluorometer (Invitrogen, USA). The average insert size was maintained at 300 ± 50 bp. Finally, paired-end sequencing (2 × 150 bp) was performed on an Illumina HiSeq platform, according to the manufacturer’s protocol (Illumina, USA).

### RNA-seq data analysis

The raw data in FASTA format was initially processed using fastp software^1^ to generate clean reads by removing adapters, poly-N sequences, and low-quality reads. Quality metrics such as Q20, Q30, and GC content were assessed, and all subsequent analyses were conducted on the clean data. To align the reads with the reference genome of *P. rhodozyma* CBS6938 (Genebank No. GCA_014706385.1), the HISAT2 tool^2^ was utilized. The expression levels of all transcripts were estimated using StringTie and quantified as Fragments per Kilobase of transcript per Million mapped reads (FPKM). Differentially expressed genes (DEGs) were identified using a fold change greater than 1.5 or less than 0.6, along with a *p*-value from the parametric F-test comparing nested linear models less than 0.05. This analysis was carried out with the edgeR package.^3^ To explore the biological relevance of these DEGs, Gene Ontology (GO) enrichment analysis was performed using the GOseq R package (Li and Dewey, 2011). Furthermore, to identify potential pathways related to the DEGs, KOBAS software was used to map significant DEGs to the KEGG pathways^4^ (Mao et al., 2005).

### Quantitative real-time reverse transcription PCR (RT-PCR)

To validate the expression levels of 26 selected DEGs identified from the transcriptomic analysis, quantitative Reverse Transcription PCR (qRT-PCR) was performed based on our previous study (Jia et al., 2024). The gene-specific primers are listed in Supplementary Table S1. The ratios of expressional levels for specific DEG in the mutant to the WT strain were analyzed using RT-PCR to evaluate the expression level of the gene in the mutant.

### Construction of the vector for gene overexpression

The overlap PCR method was utilized to construct the vector, following the protocols described by Yu et al. and our previous study (Yu et al., 2020; Jia et al., 2024). For single DEG expression, 18sup, Pgdp, G418, Tgdp, Padh4, DEG, Tact, and 18sdown were amplified separately and then ligated to the final vector for inserting into the genomic 18SrDNA site by homologous recombination for single-DEG expression (Hara et al., 2014). For dual-DEG expression, 18sup, Pgdp, G418, Tgdp, Padh4, DEG1, Tact, Padh4, DEG2, Tact, and 18sdown were amplified separately, ligated into three larger fragments, and then ligated into the final vector for dual-DEG expression. The oligonucleotide primers for constructing these vectors are listed in Supplementary Table S2.

### Genetic transformation of *P. rhodozyma* cells

The transformation of *P. rhodozyma* cells was carried out using the electrotransformation method (Niklitschek et al., 2008). Yeast cells were cultured on YM medium until the optical density at 660 nm (OD_660_) reached ~1.5. The cells were then harvested by centrifugation at 10,000 *g* for 5 min, and the resulting pellet was resuspended in a potassium phosphate buffer (50 mM, pH 7.0) and 25 mM dithiothreitol (DTT). The cell suspension was incubated at 21°C for 15 min, followed by two washes with Sucrose-Tris-Magnesium (STM) buffer (270 mM sucrose, 10 mM Tris–HCl, 1 mM MgCl_2_, pH 7.5). The washed cells were then resuspended in 500 μL STM buffer at 4°C, making them competent for transformation. For the transformation, 60 μL of competent cells were mixed with 10 μg of the DNA fragment and gently mixed. Electroporation was carried out using a Gene Pulser (BioRad, USA) under the conditions of 25 μF, 1,000 *Ω*, and 800 V.

### Validation of DEGs’ integrations into genome and their expressions

The YM medium plates with 50 mg/L G418 were used to first screen the mutants due to the G418-resistance gene in the expression vector. Genomic DNA from both the wild-type strain (WT) and the mutants was isolated as the template to validate the integrations of vectors into the 18SrDNA site by PCR. For single-DEGs’ vector confirmation, a ~ 4,500 bp DNA fragment was amplified in the positive mutants. For dual-DEGs vector confirmation, a ~ 800 bp DNA fragment was amplified in the positive mutants. The primers are listed in Supplementary Table S1. RT-PCR was used to validate the expression levels of DEGs in mutants and performed as described above.

## Results and discussion

### Nitrogen deficiency enhances astaxanthin biosynthesis while inhibiting cellular growth

**Figure 1:**
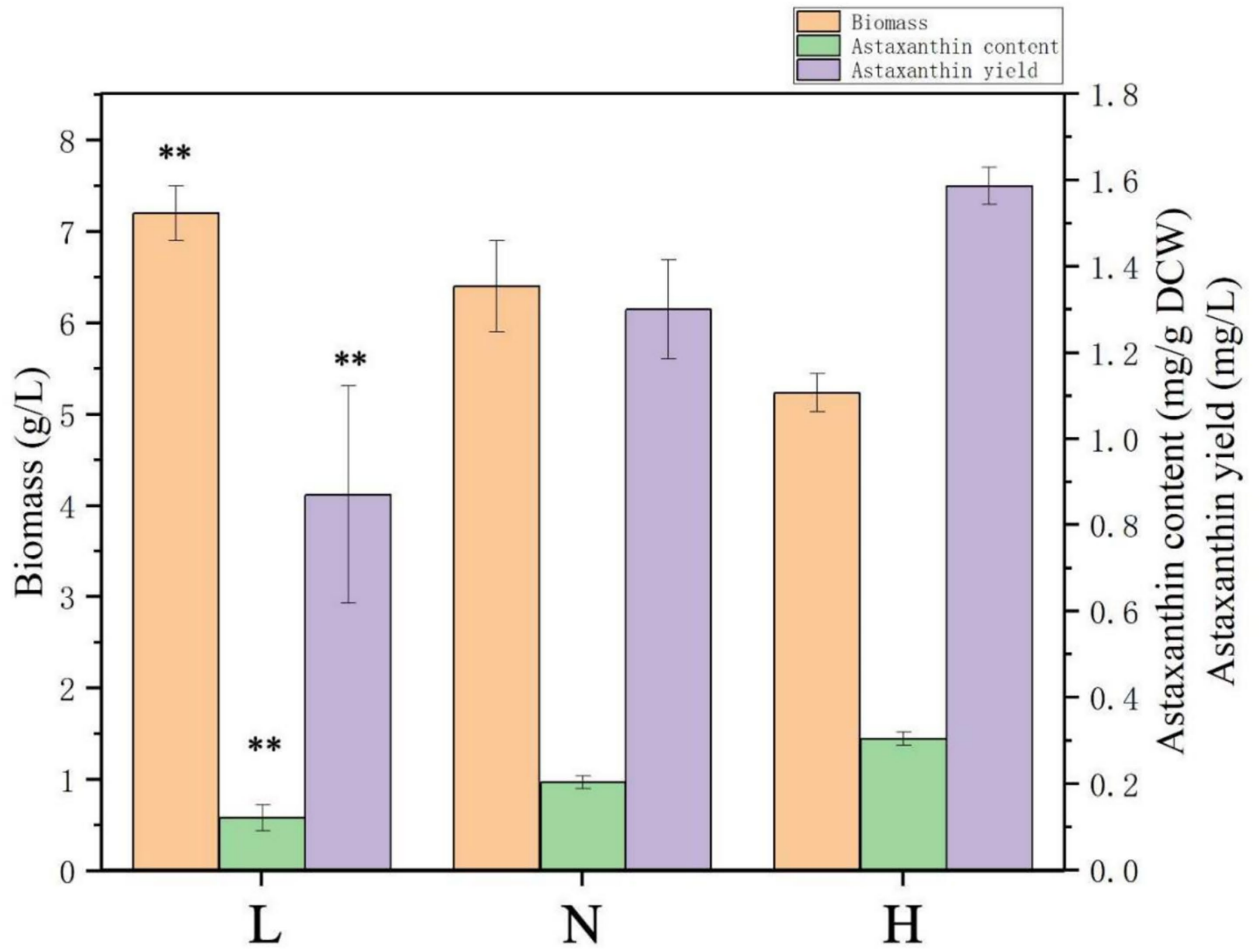
Effects of nitrogen levels in medium on biomass and astaxanthin accumulation in *Phaffia rhodozyma*. L, the medium with lower C/N ratio (~5, nitrogen-sufficiency); N, the medium with normal C/N ratio (~7.5); H, the medium with higher C/N ratio (~33, nitrogen-deficiency). Data are given as means ± SD, *n* = 3. Significance in the L group represents the comparison between the groups H and L. * *p* < 0.05, ** *p* < 0.01.

In the previous study, the YM medium with a C/N ratio of ~1.625 was used to ferment *P. rhodozyma* to produce astaxanthin, and the biomass, astaxanthin content, and yield were ~5.6 g/L, 0.2 mg/g DCW, and 0.9 mg/L, respectively (Jia et al., 2024). In this study, to analyze the effects of the C/N ratio in medium on astaxanthin fermentation, the carbon sources in YM medium were increased to 10 g/L malt extract and 50 g/L glucose, while the nitrogen sources were 5 g/L tryptone and 3 g/L yeast extract, the same as those of YM medium. The C/N ratio of this medium was ~7.5, and the medium was marked as N (normal C/N ratio). Under the N medium, the biomass, astaxanthin content, and yield were 6.4 g/L, 0.2 mg/g DCW, and 1.3 mg/L, respectively, 14.1, 50, and 111.1% higher compared with those in the YM medium (Figure 1). This result indicated that *P. rhodozyma* could tolerate higher concentration of carbon resource to convert to higher biomass. In the high-density fermentation industry, the tolerance of microbial strains to highly concentrated carbon sources is an important characteristic that is considered to determine the fermentation biomass and, consequently, the yield of intracellular products in the fed-batch strategy. Various industrial strains, such as the yeast *S. cerevisiae*, *Y. lipolytica,* and *Komagataella phaffii*, and the protist *Aurantiochytrium* sp., can utilize higher than 100 g/L, or even hundreds g/L of carbon source, to achieve high-density fermentation for efficient synthesis of high-value products (Yu et al., 2015; Xu et al., 2020; Jiao et al., 2022). For *P. rhodozyma*, a number of media optimizations were performed for efficient astaxanthin production; however, concentrations of carbon source in these media were too low to achieve high-density fermentation, resulting in potentially higher astaxanthin content but lower yields (Mussagy et al., 2023). Thus, the *P. rhodozyma* strain in this study has the potential for commercial production of astaxanthin, and screening and modification of strains tolerant to high concentrations of carbon sources is a target in future research.

To further investigate the effect of nitrogen source content on biomass and astaxanthin synthesis, based on the N medium, the nitrogen sources tryptone and yeast extract changed into 1 g/L and 0.8 g/L to form the H medium with a higher C/N ratio of ~33 and into 8 g/L and 4 g/L to form the L medium with a lower C/N ratio of ~5. As shown in Figure 1, under the L medium, biomass could reach up to 7.2 g/L, which was 38.5% higher than that in the H medium (5.2 g/L); however, the astaxanthin content and yield in H medium were 0.3 mg/g DCW and 1.9 mg/L, 150 and 118.4% higher than those in the L medium (0.12 mg/g DCW and 0.87 mg/L), respectively (Figure 1). These results showed that the nitrogen deficiency condition could enhance astaxanthin biosynthesis efficiency (astaxanthin content), which is the most important fermentation parameter, while it inhibited cellular growth, which affected the astaxanthin yield. Different types of microbial cells, including yeast, microalgae, and protist, respond to nitrogen deficiency by producing a number of high-value products, such as lipids, carbohydrates, polyunsaturated fatty acids, and terpenoids (Yu et al., 2015; Nagappan et al., 2020; Jiao et al., 2022). A two-step culturation with the first stage for cellular growth and the second stage for product synthesis is a commonly used strategy to resolve the problem of impaired cell growth when products are synthesized efficiently (Sun et al., 2017). Nitrogen deficiency conditions can lead to the accumulation of large amounts of reactive oxygen species (ROS) in cells, while the synthesis of strong antioxidant astaxanthin is one of the cellular responses to oxidative stress. Thus, in *H. pluvialis*, nitrogen deficiency could enhance the astaxanthin synthesis (Yu et al., 2022). For *P. rhodozyma*, physical and chemical modulators, such as blue light, glutamic acid, pyruvate, sodium gluconate, citric acid, penicillin, ethanol, and fluconazole, were used to increase astaxanthin productivity (Stachowiak, 2013; Li et al., 2022; Li et al., 2025). However, the effect of nitrogen level in medium on biomass and astaxanthin synthesis in *P. rhodozyma* has not yet been studied in depth, and the mechanism of nitrogen balancing the cellular growth and astaxanthin biosynthesis is still unclear. Thus, transcriptomes of the *P. rhodozyma* cells from the H and L medium are compared to illuminate the molecular mechanism of the nitrogen deficiency condition, enhancing astaxanthin biosynthesis while inhibiting cellular growth.

### RNA-seq based transcriptomic analysis

As the *P. rhodozyma* reference genome (NCBI number GCA_001007165.2_Xden1) has been released, a referential comparative transcriptome strategy was applied to illuminate the molecular mechanism of nitrogen deficiency condition, enhancing astaxanthin biosynthesis while inhibiting cellular growth (Sharma et al., 2015). The data of samples from H and L medium (H v.s. L) were pretreated with low-quality sequences removed, and the resulting data have sufficient reliability and quality for further analysis. Then, the transcriptomic reads were mapped into the reference genome of *P. rhodozyma*, and a total of 6,249 genes were identified, comparable to the genes identified in the reference genome (Sharma et al., 2015). The results demonstrated the reliability and global nature of transcriptomic data for further analyses.

**Figure 2:**
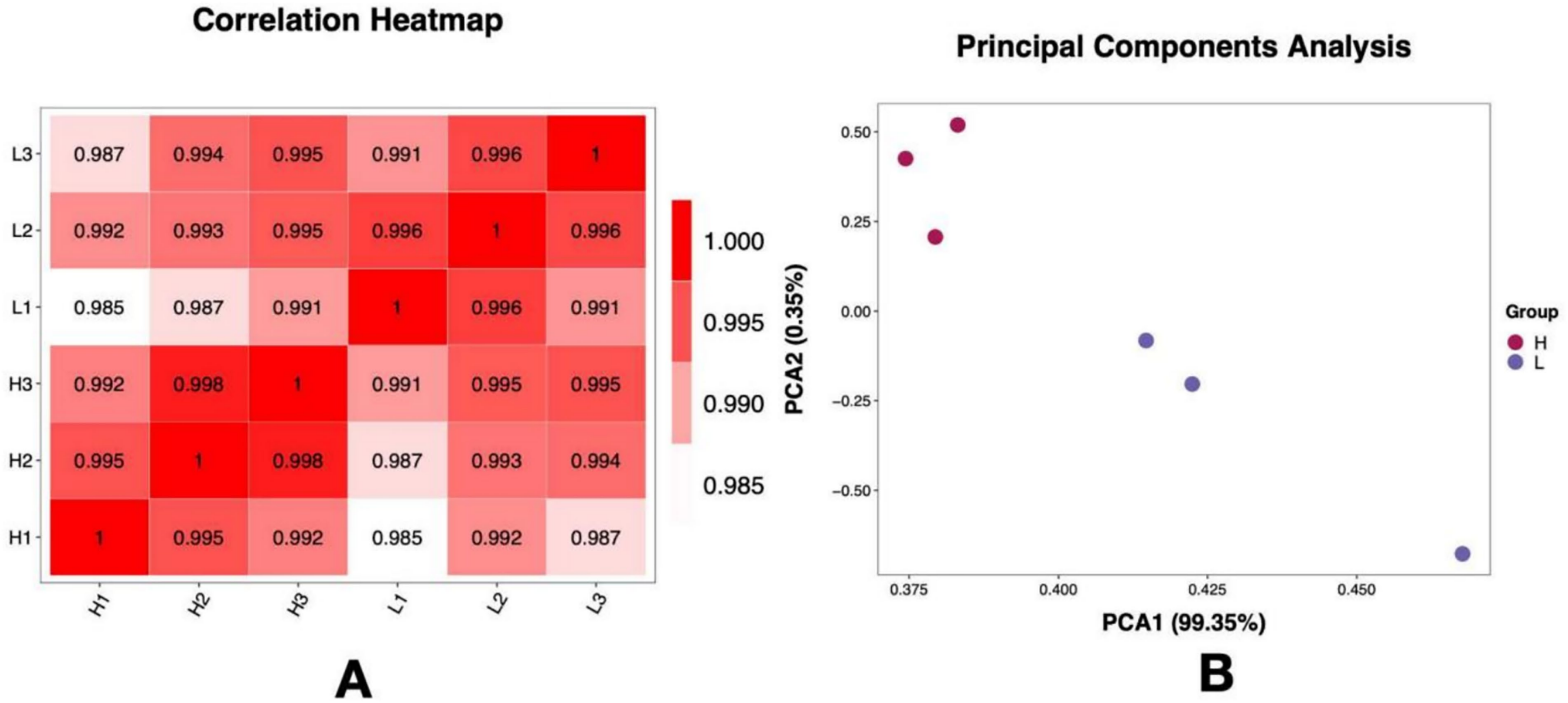
Transcriptomic profiles of the *Phaffia rhodozyma* cells under the nitrogen-deficient (high C/N ratio, H group) and nitrogen-sufficient (low C/N ratio, L group) conditions. **(A)** Pearson correlation between the samples from the H and L groups. **(B)** Principal Components Analysis (PCA) of the samples from the H and L groups.

As shown in Figure 2, the Pearson correlation analysis and PCA showed that the transcriptomic profiles of the cells from the H medium (nitrogen-deficiency condition) and the L medium (nitrogen-sufficiency condition) were clearly distinguished, indicating that changes in the level of nitrogen sources in the medium can perturb the global transcriptional profile of cells. As shown in the above results, the nitrogen-deficient (H group) condition corresponded to higher astaxanthin production and lower biomass, while the opposite was true for the nitrogen-sufficient (L group) condition; thus, further identification of DEGs between the H and L groups could elucidate the molecular mechanisms of the nitrogen-deficient condition, inducing astaxanthin synthesis and inhibiting cell growth.

### Identification, annotation, and pathway enrichment of the DEGs

**Figure 3:**
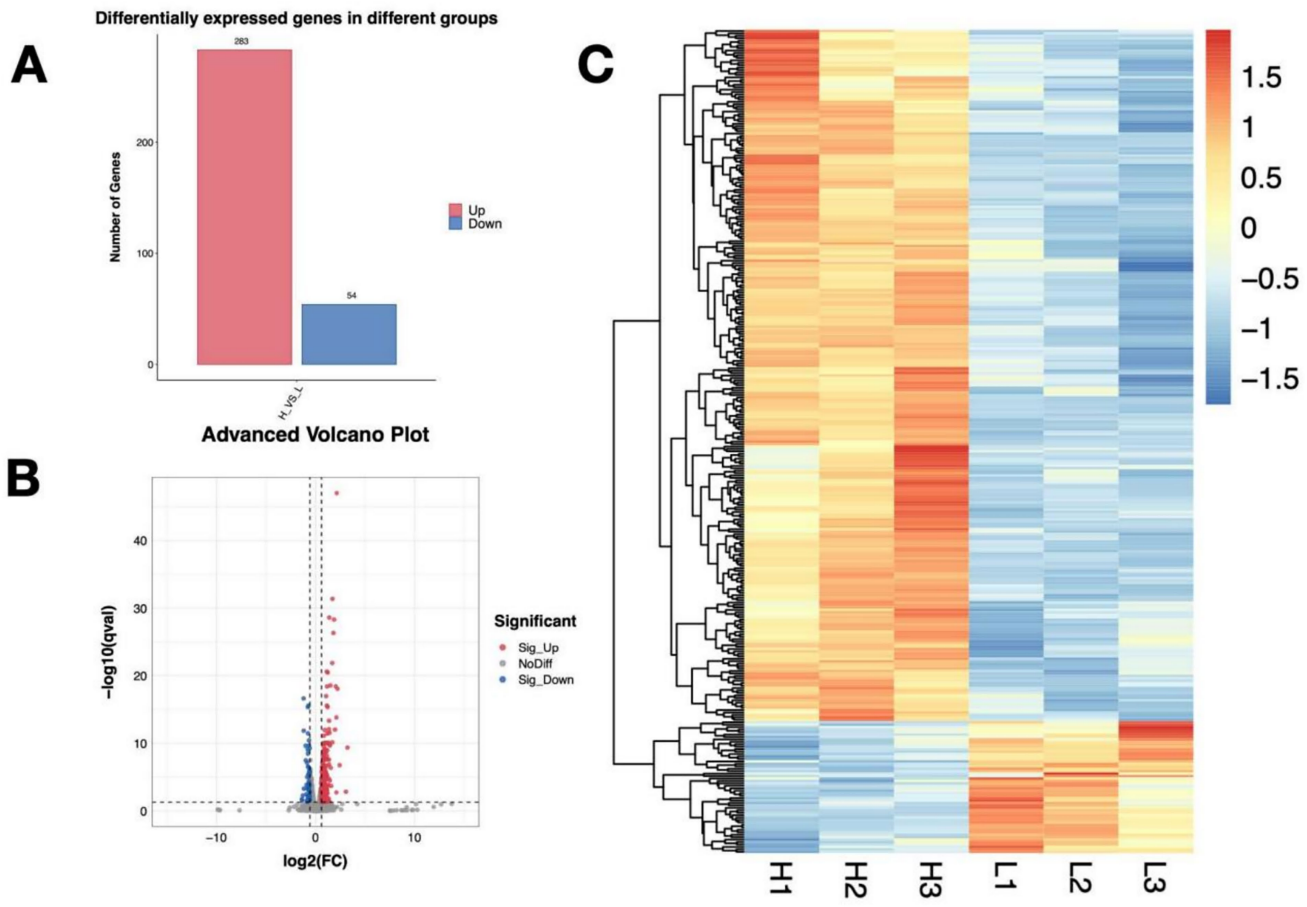
The distribution of differentially expressed genes (DEGs) between *P. rhodozyma* cells from the H medium (nitrogen-deficient) and L medium (nitrogen-sufficient). **(A)** DEGs’ number; **(B)** Volcano plot of DEGs based on log2 (FC) values; **(C)** Heatmap of DEGs based on their relative expression levels.

**Figure 4:**
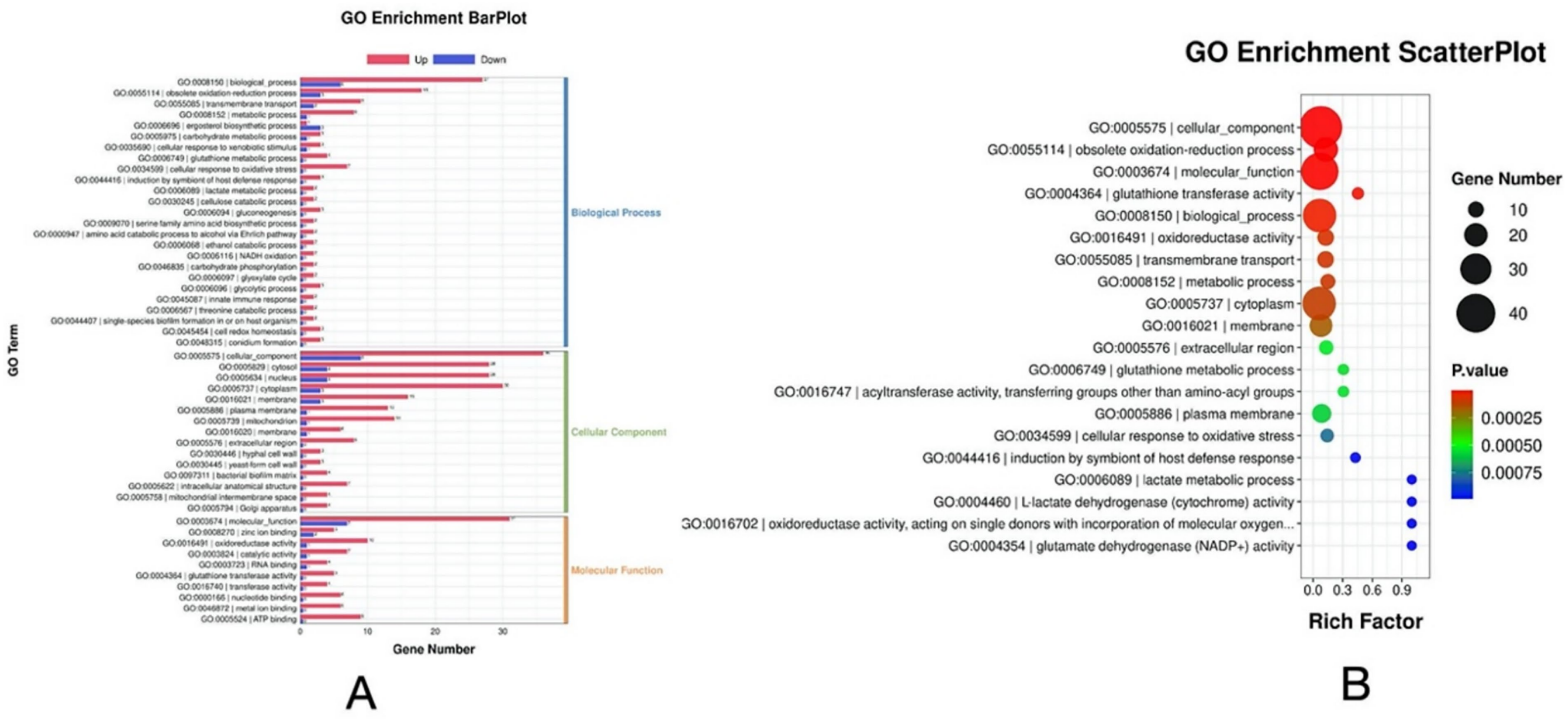
GO annotation of the DEGs between *P. rhodozyma* cells from the H medium (nitrogen-deficient) and L medium (nitrogen-sufficient). **(A)** GO enrichment bar plot; **(B)** GO enrichment scatter plot.

**Figure 5:**
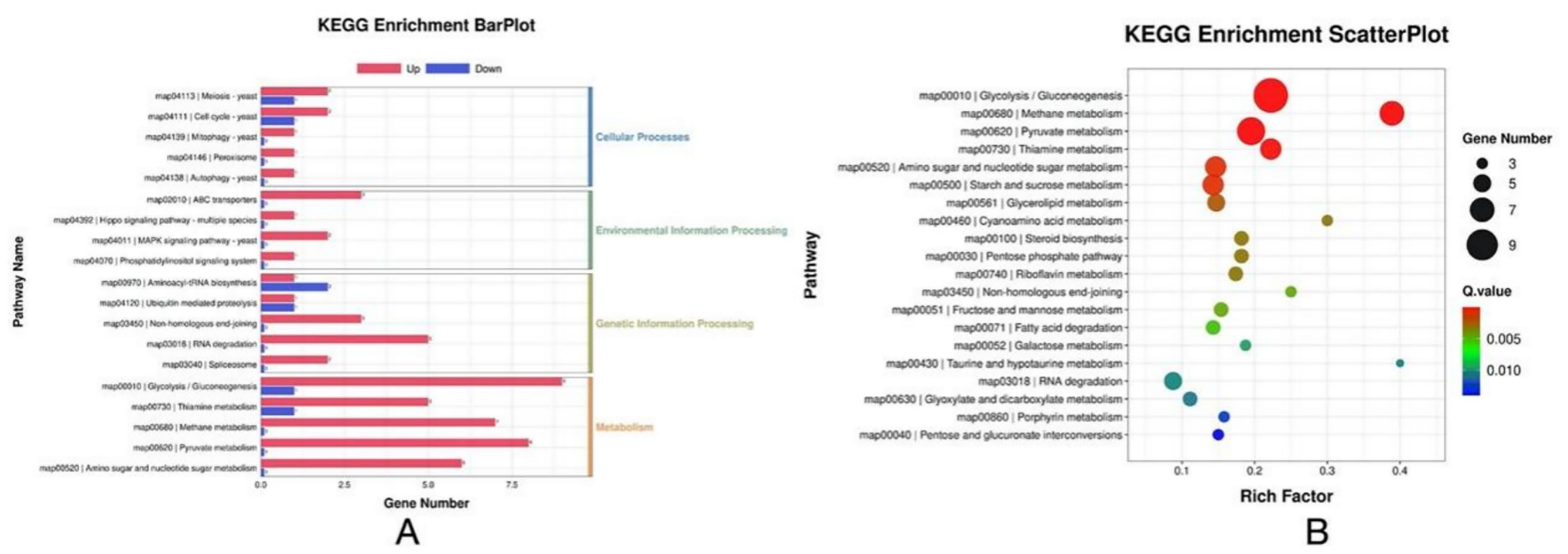
KEGG annotation of the DEGs between *P. rhodozyma* cells from the H medium (nitrogen-deficient) and L medium (nitrogen-sufficient). **(A)** KEGG enrichment bar plot; **(B)** KEGG enrichment scatter plot.

Based on the DEG’s criteria, a total of 337 DEGs with 283 upregulated and 54 downregulated were identified in the *P. rhodozyma* cells from the H and L groups (Figure 3A). The distribution of these DEGs and their clustering relations based on their relative expression levels were further analyzed (Figures 3B,C). To study the function of these DEGs, they were further enriched into the Gene Ontology (GO) and the Kyoto Encyclopedia of Genes and Genomes (KEGG), respectively. As show in Figure 4, DEGs were mainly enriched into 50 GO terms under three main domains, in which the GO terms “cellular component,” “oxidation–reduction process,” glutathione transferase,” “transmembrane transport,” “glutathione metabolic process,” “lactate metabolic process,” “glutamate dehydrogenase (NADP+) activity,” “cellular response to oxidative stress,” and “induction by symbiont of host defense response” were most enriched, indicating that the DEGs were mainly involved in cellular resistance, amino acid metabolism, redox stress, and substance transport (Figure 4). For KEGG enrichment analysis, the DEGs were mapped to 19 KEGG terms across four major domains, with the most enriched terms being “glycolysis,” “pyruvate, methane, thiamine, riboflavin metabolism,” “steroid biosynthesis” “pentose phosphate pathway,” “non-homologous end-joining,” “fatty acid degradation,” “ABC transporter,” and “MAPK signaling,” indicating that DEGs were mainly involved in central carbon and nitrogen metabolism, cellular repair, transporting, and signal pathways (Figure 5).

The annotations of DEGs were further analyzed to uncover their associations with cell growth and astaxanthin synthesis in *P. rhodozyma*. Unexpectedly, DEGs were not within the astaxanthin biosynthesis pathway, indicating that the nitrogen-deficient condition enhances astaxanthin synthesis or inhibits cellular growth by global regulation of genes outside the astaxanthin synthesis pathway (Table 1). A total of 26 DEGs from six categories were selected to be analyzed and verified through RT-PCR. The results from RT-PCR had a similar trend to those from transcriptome data (FPKM values), indicating that the transcriptome data are sufficiently reliable for subsequent analysis (Table 1). Enolase, phosphoenolpyruvate carboxykinase, and citrate synthase involved in glycolysis, and maltase glucoamylase used to metabolize maltose were increased in the nitrogen-deficient condition. Since glucose and maltose are the main carbon sources in the medium, this result indicated that the nitrogen-deficient condition accelerated the metabolic utilization of carbon sources in *P. rhodozyma*, dissociating the glucose effect and providing more carbon sources for astaxanthin synthesis (Torres-Haro et al., 2021). Gluconate kinase in the pentose phosphate pathway and isocitrate lyase in the glyoxylate cycle are the main producers of reducing power NADPH, and their increased expression levels result in increased astaxanthin synthesis without DEGs in the astaxanthin synthesis pathway. In addition, malate dehydrogenase and 2-oxoglutarate dehydrogenase in the TCA cycle were significantly induced to produce more NADH and FADH_2_ for the formation of ATP (Gasmi et al., 2021), similar to the increased malate dehydrogenase activity induced by the titanium dioxide stress for astaxanthin production in *P. rhodozyma* (Zhang et al., 2021). Glycine dehydrogenase and glutamate dehydrogenase involved in amino acid metabolism and NADH formation were induced, indicating that conversion from amino acid to energy is accelerated in the nitrogen-deficient condition. Exogenous glutamate and titanium dioxide were found to stimulate nitrogen metabolism to enhance astaxanthin in *P. rhodozyma* (Yang et al., 2023; Cai et al., 2024). However, sodium orthovanadate (SOV) could promote carbon flux into the astaxanthin synthesis pathway by inhibiting glutamate dehydrogenase activity and amino acid metabolism, suggesting that nitrogen deficiency and regulator treatments regulate astaxanthin synthesis in different manners (Song et al., 2024). Meanwhile, alcohol dehydrogenase catalyzes the cytosolic reduction of pyruvate to ethanol with an oxidation of NADH to NAD^+^. The state of NADH/NAD^+^ coupled with ATP/ADP plays an important role in astaxanthin synthesis (Flores-Cotera et al., 2021). These results indicated that nitrogen deficiency regulates astaxanthin synthesis by perturbing central and energy metabolism.

**Table 1:** The DEGs explaining improved astaxanthin and impaired growth induced by nitrogen-deficiency stress.

Category	Gene ID	Gene description	FPKM		log2(fc)	RT-PCR validation
			Nitrogen-deficiency	Nitrogen-sufficiency		
Central and energy metabolism	3,063	enolase	831.9 ± 40.7*	517.9 ± 15.0	0.7	0.4
	445	phosphoenolpyruvate carboxykinase	98.8 ± 5.5*	54.3 ± 12.7	0.9	1.3
	5,511	citrate synthase	24 ± 0.4*	14.9 ± 3.1	0.7	0.5
	3,959	Maltase glucoamylase	339.1 ± 37.9**	105.2 ± 0.1	1.7	2.1
	8,675	Gluconate kinase	33.0 ± 4.8*	17.9 ± 3.1	0.9	0.5
	10,441	isocitrate lyase	8.3 ± 0.8	4.9 ± 0.9	0.8	1.2
	12,519	malate dehydrogenase	5.9 ± 0.4**	3 ± 0.3	1	0.6
	7,463	alcohol dehydrogenase	54.2 ± 3.2**	21.4 ± 7.3	1.3	0.9
	11,397	glutamate dehydrogenase	63.9 ± 3.9**	29.5 ± 1.6	1.1	1.5
	1,851	glycine dehydrogenase	14.8 ± 0.1*	8.5 ± 1.3	0.8	1
	983	2-oxoglutarate dehydrogenase	82.8 ± 3.4*	53.6 ± 1.5	0.6	0.8
Anti-stress	1,635	Iron/ascorbate family oxidoreductases	28.8 ± 2.6*	17.3 ± 2.0	0.7	0.6
	2,145	Mitochondrial sulfhydryl oxidase	180 ± 9.1*	108.5 ± 7.1	0.7	0.8
	4,669	Thioredoxin	88.4 ± 15.5*	49.8 ± 6.1	0.8	0.6
	2,521	NADH-cytochrome b-5 reductase	8.6 ± 1.0*	4.8 ± 0.8	0.9	1.2
	8,101	hypothetical protein	782.4 ± 45.3	518.9 ± 16.8	0.6	0.9
Signal transduction	3,687	SHO1	9.2 ± 0.4*	5.9 ± 1.1	0.6	0.9
	4,281	srf-type TF	5 ± 0.3*	2.7 ± 0.4	0.9	0.9
	8,655	serine/threonine kinase	2.6 ± 0.3*	1.5 ± 0.4	0.8	1.3
	9,861	calcium calmodulin-dependent protein kinase	22.8 ± 0.1*	13.1 ± 2.7	0.8	0.5
Another metabolism	2,737	Sterol reductase	6.1 ± 0.5*	9.7 ± 0.5	−0.6	−0.8
	2,739	C-24 reductase	7.2 ± 1.0*	11.0 ± 2.2	−0.6	−0.3
Fatty acid metabolism	11,631	aldehyde dehydrogenase	557.1 ± 21.1*	348.4 ± 94.6	0.7	0.5
Transportation	4,011	ABC transporter	61.6 ± 3.2*	38.5 ± 3.7	0.7	0.5
	1,059	Ferric reductase	3.4 ± 0.2*	5.5 ± 1.3	−1	−0.9
	10,279	dicarboxylic amino acid permease	113 ± 4.4*	172.2 ± 12.5	−0.6	−1

Data are given as means ± SD, *n* = 3.

*p < 0.05.

**p < 0.01.

The DEGs iron/ascorbate family oxidoreductases, mitochondrial sulfhydryl oxidase, thioredoxin, and NADH-cytochrome b-5 reductase are antioxidant genes for cellular response to oxygen stress, and their increased expression levels, coupled with the results mentioned above, indicated that nitrogen deficiency leads to NADH/NAD^+^ imbalance and energy metabolism perturbation to produce excessive ROS (Flores-Cotera et al., 2021). *P. rhodozyma* either lacks or possesses lower cytosolic superoxide dismutase (Cu/Zn SOD, and SOD1) and catalase activities compared with *S. cerevisiae*; thus, this yeast depends primarily on astaxanthin for its antioxidant defense, and oxidative stress is the most common strategy for astaxanthin production (Schroeder and Johnson, 1993; Zhang et al., 2021; Mussagy et al., 2023). In this study, the results indicated that the mechanism of *P. rhodozyma* cells responding to oxygen stress is more complex, involving not only astaxanthin synthesis but also the antioxidant/reactive oxygen scavenger genes, suggesting more potential target genes for modifying this yeast to overproduce astaxanthin. A hypothetical protein (Gene No. 8101) annotated as a non-homologous end-joining (NHEJ) protein for DNA double-strand break repair was induced significantly by the nitrogen-deficient condition (Table 1), suggesting that nitrogen-deficiency stress causes breaks in the genomic DNA, and the NHEJ system is activated to enable *P. rhodozyma* cells survive in this stress condition. *P. rhodozyma* is a homologous recombination dominant yeast and benefits homologous-recombination-based genomic modification and unstable non-homologous recombination-based plasmid expression, while in NHEJ yeasts, the NHEJ proteins can only be activated for more efficient genetic manipulation (Zhang et al., 2019). However, the result in this study suggested that the NHEJ system was induced by stress conditions for DNA repair and cellular survival; in other words, damaged cell growth is associated with the NHEJ system. Thus, the NHEJ system is a potential genetic target for constructing astaxanthin-overproduction strains.

Sterol reductase and C22-sterol desaturase in the sterol metabolism pathway were significantly inhibited by the nitrogen-deficient condition, indicating that this stress can inhibit the competitive pathways diverted from farnesyl pyrophosphate (FPP) with astaxanthin synthesis to improve astaxanthin production (Shi et al., 2022). Mutagenized and melatonin-, TiO_2_-, and ethanol-treated *P. rhodozyma* strains with higher astaxanthin productivity had weakened the sterol synthesis pathway, confirming a competition between sterol and astaxanthin synthesis (Gu et al., 1997; Shi et al., 2022; Jia et al., 2024). As another competitive pathway to astaxanthin synthesis, fatty acid biosynthesis competes for acetyl-CoA to synthesize the carbon skeleton. The *P. rhodozyma* mutants or stress-treated strains with higher astaxanthin productivity have lower fatty-acid-synthesis activity to increase more carbon flux to astaxanthin synthesis (Miao et al., 2011; Jia et al., 2024). As shown in Table 1, aldehyde dehydrogenase in fatty acid degradation was increased by the nitrogen-deficient condition to form more acetyl-CoA for astaxanthin synthesis, realizing carbon flux to astaxanthin synthesis from another perspective. In contrast, TiO_2_ could increase fatty acid synthesis to enhance esterification of astaxanthin and hence its productivity (Zhang et al., 2022). This result shows that fatty acids and astaxanthin synthesis have a complex relationship, and the balance between them is important for astaxanthin productivity.

The ABC transporter maintains the balance between accumulation and transmembrane transport of the secondary metabolites. Astaxanthin is not automatically released from the cells; thus, its accumulation in the cells causes cellular toxicity. Overexpression of the ABC transporter gene promoted the secretion of carotene in *S. cerevisiae* (Bu et al., 2020). Induction of ABC transporter by nitrogen deficiency in this study could reduce toxicity caused by astaxanthin accumulation in cells (Table 1). Dicarboxylic amino acid permease is responsible for aspartic acid’s and glutamic acid’s absorption and is specifically induced in response to the presence of its substrate in the medium (Horák, 1997). In the nitrogen-deficient medium, the dicarboxylic amino acid permease was inhibited due to the reduced availability of the substrate in the medium, which led to impaired cellular growth (Table 1). Ferric reductase, as a membrane protein, reduces extracellular ferric iron to biologically available ferrous iron and plays an essential role in high-affinity iron acquisition. In addition, due to a close mutual relationship between yeast cellular iron homeostasis and oxidative stress, ferric reductase plays an important role in oxidative stress response. In the yeast *Candida albicans*, the ferric reductase–deficient mutants were more sensitive to the oxidants H_2_O_2_ and menadione, suggesting that the reaction of intracellular iron with hydrogen peroxide via the Fenton reaction to form radicals is highly toxic (Xu et al., 2014). As shown in Table 1, decreased ferric reductase by nitrogen deficiency alters intracellular iron homeostasis in *P. rhodozyma*, which, in turn, alters the cellular response to oxidative stress caused by nitrogen deficiency, as the above results, thereby affecting cell growth.

SHO1, serine/threonine kinase, and srf-type transcription factor (TF) are the components in the mitogen-activated protein kinases (MAPKs) signal pathway, while calcium calmodulin–dependent protein kinase is the essential component in the Target of Rapamycin (TOR) signal and calmodulin signal pathways (Jia et al., 2024). Their increased expression levels by nitrogen deficiency showed that this stress enforces the MAPKs, TOR, and calmodulin signal pathways, thereby enhancing the regulation of these pathways on downstream target genes, which, in turn, regulates cell growth and astaxanthin synthesis in *P. rhodozyma*.

### Overexpression of DEGs individually or synergistically for astaxanthin synthesis

**Figure 6:**
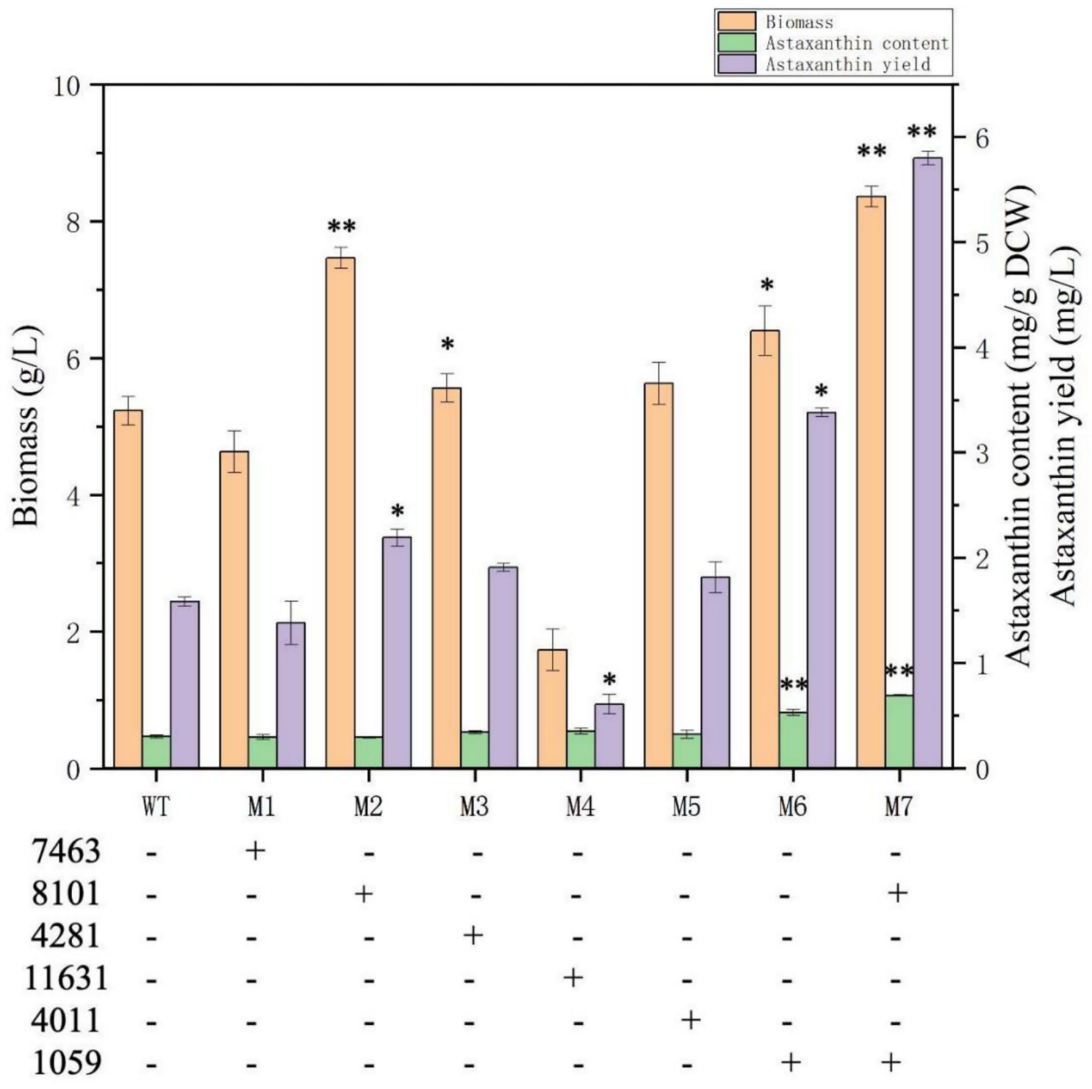
Biomass, astaxanthin content, and yield of *P. rhodozyma* mutants individually and synergistically expressing DEGs under nitrogen-deficient condition. 7,463, alcohol dehydrogenase; 8,101, hypothetical protein (NHEJ gene); 4,281, srf-type TF; 11,631, aldehyde dehydrogenase; 4,011, ABC transporter; 1,059, ferric reductase. Data are given as means ± SD, *n* = 3. Significances in mutants represent the comparisons between the mutants and wild strain (WT). * *p* < 0.05, ** *p* < 0.01.

To further validate the functions of the DEGs identified above and to construct astaxanthin-overproducing mutants, the DEGs alcohol dehydrogenase (energy, No.7463), hypothetical protein (NHEJ gene for DNA repair, No.8101), srf-type TF (global regulation, No. 4281), aldehyde dehydrogenase (degradation of competing metabolites, No.11631), ABC transporter (metabolite effluent and detoxification, No.4011), and ferric reductase (iron absorption and antioxidant, No.1059) were selected to be overexpressed individually and synergistically in *P. rhodozyma* wild strain for balancing the cellular growth and astaxanthin production under nitrogen-deficiency stress. The individual-gene-overexpressing plasmids for more than six DEGs with 18SrDNA as the insertion site in the genome were constructed and transformed into the *P. rhodozyma* strain AS2.1557 (WT), and the mutants were randomly obtained, respectively. The ~4,500-bp fragments specific for six single DEG vectors and the ~800-bp fragments for the DEGs 8,101 and 1,059 were amplified in the positive *P. rhodozyma* mutants but not amplified in the *P. rhodozyma* WT strain, indicating that the six single-DEG expression vectors and the dual-DEGs expression vector were integrated into the genome of related mutants (Supplementary Figure S1). The relative expression levels of the six DEGs in their mutants compared with the WT strains showed that the six DEGs were overexpressed individually and synergistically in the mutants 1–6 and mutant 7, respectively (Supplementary Table S3). As shown in Figures 1, 6, the biomass of the mutant M2 expressing the NHEJ gene for DNA repair could reach 7.5 g/L, which was 44.2% higher than that in the WT strain under the nitrogen-deficient and was the same as the WT strain under the nitrogen-sufficient condition. The improved cellular growth resulted in a 37.5% increase in astaxanthin yield with unchanged content. The result indicated that the DNA break caused by the nitrogen-deficient stress leads to impaired cell growth, and the induced NHEJ gene for DNA repair is insufficient to restore the cellular growth in the nitrogen-sufficient condition. Thus, additional overexpression of the NHEJ gene under nitrogen-deficient conditions resumes their growth. In *S. cerevisiae*, DNA double-strand breaks caused by oxidative stress can be repaired through the NHEJ system; however, genetic modification of cellular resistance to stress for efficient microbial production based on the NHEJ system is rarely reported (Letavayová et al., 2006). Genetic modification to balance microbial NHEJ and homologous recombination (HR), anti-stress, and genetic efficiency is a future research perspective. In the mutant M6 expressing the ferric reductase gene, its biomass and astaxanthin content were 6.4 g/L and 0.53 mg/g DCW, which were 23.0 and 76.7% higher than those in WT strain under the nitrogen-deficient condition, resulting in an 112.5% increasement in astaxanthin yield (Figure 6). In *S. cerevisiae*, heterologous production of carotenoids induces ferric reductase gene and leads to a decrease in cell membrane (CM) fluidity and deficiency in metal ions; thus, ferric reductase and carotenoids synthesis are closely related (Liu et al., 2016). In *P. pastoris*, overexpression of ferric reductase increases cellular tolerance to lactate and thus lactate production (Sae-Tang et al., 2023). A ferric reductase-deficient *C. albicans* mutant showed more sensitivity to oxidative stress, and ferric reductase might be a potential target for antifungal drug development (Xu et al., 2014). In the mutant M4, in which the aldehyde dehydrogenase gene was overexpressed, its biomass was severely inhibited, being 67.3% lower than that in the WT strain, and its astaxanthin yield was 62.5% lower than the WT strain. Although aldehyde dehydrogenase has cellular detoxification and fatty acid oxidation to provide more acetyl-CoA for astaxanthin synthesis, its overexpression might cause dysregulation of cellular energy and fatty acid metabolism, resulting in impaired cell growth (Kwolek-Mirek et al., 2024). For alcohol dehydrogenase (M1), srf-type TF (M3), and ABC transporter (M5), their overexpression did not significantly affect biomass and astaxanthin synthesis (Figure 6), indicating the diversity of regulatory mechanisms.

To verify whether multigene expression is synergistic and to further increase astaxanthin production, the NHEJ gene for DNA repair (No.8101) and the ferric reductase (No.1059) were integrated into the dual-DEG expression vector and transformed to the *P. rhodozyma* WT strain to obtain the mutant M7. As shown in Figure 6, the biomass and astaxanthin content of M7 were significantly increased compared with the mutants M2 and M5, with improvements of 61.5 and 133.3%, respectively, under nitrogen-deficient conditions. The result indicates that the NHEJ gene for DNA repair and ferric reductase, which promote cellular growth and astaxanthin synthesis, respectively, work synergistically to balance growth and astaxanthin production under nitrogen-deficienct stress, leading to more efficient astaxanthin synthesis.

**Figure 7:**
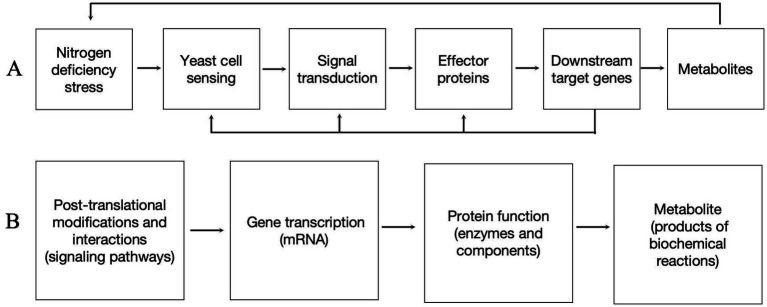
Molecular mechanisms of yeast cell response to nitrogen deficiency. **(A)** Yeast response to nitrogen deficiency stress. **(B)** Different molecular levels occurring in the mechanisms.

Based on our findings, the DEGs induced by nitrogen-deficienct stress in *P. rhodozyma* involve not only enzymes from individual metabolic pathways but also signaling pathways and transcription factors (TFs). For the first time, we propose a model for the regulation of cellular growth and astaxanthin synthesis in P. rhodozyma under nitrogen deficiency: cells sense changes in nitrogen sources in the medium, triggering signaling pathway alterations that activate effector proteins, such as transcription factors, which then regulate multiple downstream target genes. The molecular regulation occurs sequentially at the level of protein post-translational modifications and interactions (signaling pathways), gene transcription (mRNA), protein function (enzymes and components), and metabolite production (products of biochemical reactions). Additionally, the primary types of regulated genes are involved in central and energy metabolism, stress responses, material absorption and transport, and competitive pathways related to astaxanthin synthesis, but not directly in the astaxanthin synthesis pathway itself. This finding suggests that the regulation of nitrogen-deficient stress is complex and operates at a global cellular level (Figure 7). Our results provide new targets for the genetic modification of *P. rhodozyma* to enhance astaxanthin production. Future studies will validate the functions of these DEGs and further investigate their relationship with astaxanthin synthesis.

In this study, a total of six DEGs were overexpressed in the *P. rhodozyma* wild-type strain. However, only the NHEJ gene, involved in DNA repair, and ferric reductase showed positive effects on cellular growth and astaxanthin synthesis. These results suggest that a 0.6-fold increase in NHEJ gene expression under nitrogen-deficient conditions does not fully protect the cells from oxidative stress, but further upregulation of the gene provides additional protection. Ferric reductase expression decreased by 1-fold under nitrogen deficiency, and its overexpression further enhanced astaxanthin synthesis, indicating that, despite the nitrogen—deficient conditions being beneficial for astaxanthin production, ferric reductase acts as a rate-limiting enzyme. Co-expression of these two genes displayed a synergistic effect. However, when other dual-gene combinations were co-expressed, no positive transformants were obtained (data not shown). This may be due to the lower transformation efficiency of large exogenous DNA fragments or reduced genome integration efficiency. Therefore, as the number of genetic targets increases, more efficient genetic tools, such as advanced transformation methods or the CRISPR-Cas9 system, should be developed for *P. rhodozyma*.

## Conclusion

A high carbon source concentration and a high C/Nratio (nitrogen deficiency) condition is favorable for astaxanthin synthesis but detrimental to cellular growth, often resulting in low overall astaxanthin production in *P. rhodozyma*. The regulation of astaxanthin synthesis under nitrogen deficiency occurs not directly within the astaxanthin biosynthesis pathway but at a global cellular level. A regulatory model at the molecular level is proposed to deepen the theory of regulation of yeast astaxanthin synthesis and provide more potential targets for future engineering. The synergistic co-overexpression of DNA repair and ferric transport genes in *P. rhodozyma* can effectively balance cellular growth and astaxanthin synthesis, leading to more efficient astaxanthin production. The engineered strains with enhanced astaxanthin production have significant industrial application potential. However, there are limitations to this approach, including the need for improved transformation efficiency and the optimization of co-expression systems for multiple genes. Future work should focus on enhancing the stability and scalability of these engineered strains under industrial fermentation conditions, as well as exploring other regulatory pathways that could further improve astaxanthin yield. Additionally, developing more efficient genetic tools, such as CRISPR-Cas9-based systems for precise genome editing, will help overcome current limitations and enable more targeted modifications for improved productivity.

## Data Availability

The original contributions presented in the study are included in the article/Supplementary material, further inquiries can be directed to the corresponding author.

## Footnotes

^1^ https://github.com/OpenGene/fastp

^2^ https://ccb.jhu.edu/software/hisat2

^3^ https://bioconductor.org/packages/release/bioc/html/edgeR.html

^4^Kyoto Encyclopedia of Genes and Genomes, http://en.wikipedia.org/wiki/KEGG
