# Supplementary material for: A transcriptome-guided genetic engineering strategy to balance cell growth with astaxanthin production in Phaffia rhodozyma
Source: Front Microbiol. 2025 Sep 18;16:1636554. doi: 10.3389/fmicb.2025.1636554 (PMC12489986; doi:10.3389/fmicb.2025.1636554)
Supplement: Supplementary file 1 [file Data_Sheet_1.pdf]

Supporting information

A transcriptome-guided genetic engineering strategy to balance cell growth with astaxanthin production in *Phaffia rhodozyma*

Jianping Jia [1], Chenxi Ye [1], Sainan Jin [1], Qingqing Li [1], Zhengyi Pan [1], Wen Wen [1] and Guoliang Bao [1*]

[1] School of Pharmacy, School of Food Science and Engineering, Hangzhou Medical College, Hangzhou, China

*Address correspondence to:

Guoliang Bao, E-mail: 2020000091@hmc.edu.cn

Table S1 The primers used for RT-PCR in this study

Gene	Primers
enolase	F-TCCCGAGGTAACCCCACCG
	R-AAGGCCTTGAGTCCGGGG
phosphoenolpyruvate carboxykinase	F-CTTCGTTGTCCCAAGCCGG
	R-CCTGGTTGAACATGCCAGCAAG
citrate synthase	F-TTCGTCCGATACTATTACGGTGATCG
	R-GTGAGCACGATAGTCAGATCAAAAATCAACAA
Maltase glucoamylase	F-GGCCGACTGCTCAAAACGG
	R-ATAGCACCGGTTACGAGCACAT
Gluconate kinase	F-TATTCTTGACAGCATCCGCTCTCT
	R-TCGCCGGAGGTTCCTCTATCG
isocitrate lyase	F-CGAACGAGCAGCTTCATCTCA
	R-AGCCTCGGAAACGAacTTGGAAA
malate dehydrogenase	F-CTCAGAGGTGACTTTATTCTAGACGAACCAGA
	R-GTTGGCATTCGCAAAGTTCCGT
alcohol dehydrogenase	F-CCAGAAGGCGTGGGTGTTC
	R-CACCGCCGATCTGCTTGAGA
glutamate dehydrogenase	F-CGAGCAGGCCCTTAACGAG
	R-GCTCTGGCAGAATCGTCGGATC
glycine dehydrogenase	F-GCGGTCAGCCAAACAGCA
	R-CTTGAGCAAAGTCAACGCCAACA
2-oxoglutarate dehydrogenase	F-GCTCTCAGGCCCACAGTCTC
	R-AGCCTCGATAGGTTTCCTGATGACAT

Iron/ascorbate family oxidoreductases	F-CCGAGACGTTGCACTCCGA
	R-GACTTCATGGTAGTACTTCAAGACATCACTTC
Mitochondrial sulfhydryl oxidase	F-CCTATCTTCCTCGGCCTAGCTACT
	R-CTTGACATGGCGTGAAGGAACG
Thioredoxin	F-CTCTTTCGTCTCATCTGCTGGGT
	R-GCTGTTGGTGCTTATCCCAGC
NADH-cytochrome b-5 reductase	F-CCTCTGGCTAGAGGGTTTTCTACC
	R-ACCAGGAGGGCCACAGACA
hypothetical protein	F-CACCTCCAGCCCAACCAGC
	R-GAGACCAAGGATGGTGTCAATTGC
SHO1	F-TACTCATTACATCCTCCTTATCACTTGGGC
	R-TGCTTGTTGAGGATGCAAAGAACCAAA
srf-type TF	F-ACGAGCAGAATCGTCAGCTACATC
	R-ATCCTAGGGGTTCTCCTCCGG
serine/threonine kinase	F-TGGGGAAACAAACTTGTTTCATTTCTATCTTC
	R-GTCGATCTGACAGGCATAACCCTT
calcium calmodulin-dependent protein kinase	F-CTCCTTCGGCTATCTACGGGAAAC
	R-GCTTTTTCGCAGATGAGGACGG
Sterol reductase	F-TTGCTTCTTTTATCCTACTACATTTTCGATACCG
	R-GATGATGAACCAGACAGGGTAGAAGT
C-24 reductase	F-GCAAACGGATCGTCAAATGGCC
	R-TGTACCAACAGCCGATGGCATT
aldehyde dehydrogenase	F-CGCCTTCAAGGGTGAAGTCACTA
	R-ATCTTTCCTCGTTCGTGTCCAGG
ABC transporter	F-AAGACAGCTCGAGCTGATTGC

	R-GAGGGAACCAGTGCCCCA
Ferric reductase	F-GGCAGGGCGTACAATACCAC
	R-CAATGATCATTCCTCTGCTCACCAC
dicarboxylic amino acid permease	F-ACTTTGAGAAGGACGGAGGCG
	R-ACGATGAGGTACTTGAACAGGTAGTTGT

Table S2 The primers for constructing the single- and dual-DEGs overexpression vectors

Categorization	Primers	Sequence
Common primers	18sup-F	AGTCATATGCTTGTCTCAAAGATTAAGCCA
	18sup-R	TCTCGGGTTCCCTCGACTTGTCATTACGGCGATCCTAGAAACCAA
	Pgpd-F	TTGGTTTCTAGGATCGCCGTAATGACAAGTCGAGGGAACCCGAGA
	Pgpd-R	AGCAAGACGTTTCCCGTTCATGATGGTAAGAGTGTTAGAGAAGTAGTGGTGG
	G418-F	CCACCACTACTTCTCTAACACTCTTACCATCATGAACGGGAAACGTCTTGCT
	G418-R	AGAGGGTTTGGAGAGAACCGTTTATAACCAATTCTGATTAGAAAAACTCATCGAGCA
	Tgpd-F	TGCTCGATGAGTTTTTCTAATCAGAATTGGTTATAAACGGTTCTCTCCAAACCCTCT
	Tgpd-R	CTTACACGGTCGACTTTCTTTTCTTTACCTGGAAGGGCTGCTGATGGA
	Padh4-F	TCCATCAGCAGCCCTTCCAGGTAAAGAAAAGAAAGTCGACCGTGTAAG
	Tact-R	GAATACTAATGCCCCCAACTATCCCTATTAAACCTGCCGGAGCTGAAG
	18sdown-F	CTTCAGCTCCGGCAGGTTTAATAGGGATAGTTGGGGGCATTAGTATTC
	18sdown-R	TACGGAAACCTTGTTACGACTTTTACTTCC
Primers for single-DEG	Padh4-7463-R	tgtggggttcagatgatgtgttattggCAGCACAAGACAGCGCAT
	7463-F	ccaataacacatcatctgaaccccacaATGCGCTGTCTTGTGCTGC
	7463-R	cctgccttaaaggatagaaagactttgttgatGCTGGGCCTGCAGGTG
	Tact-7463-F	CACCTGCAGGCCCAGCatcaacaaagtctttctatcctttaaggcagg
	Padh4-8101-R	ggagatcgCTCGGTGGACATtgtggggttcagatgatgtgttattgg
	8101-F	ccaataacacatcatctgaaccccacaATGTCCACCGAGcgatctcc
	8101-R	cctgccttaaaggatagaaagactttgttgatGACATCGAAGGCTTCCGATCGC
	Tact-8101-F	GCGATCGGAAGCCTTCGATGTCatcaacaaagtctttctatcctttaaggcagg
	Padh4-4281-R	ACATCATCCTCCAtatcctcctccattgtggggttcagatgatgtgttattgg
	4281-F	ccaataacacatcatctgaaccccacaatggaggaggataTGGAGGATGATGT

	4281-R	cctgccttaaaggatagaaagactttgttgatCGTACCCCATGCTCCAATATCCT
	Tact-4281-F	AGGATATTGGAGCATGGGGTACGatcaacaaagtctttctatcctttaaggcagg
	Padh4-11631-R	GGTCAAACTTTTTAGTGAACGTTTTGGACATtgtggggttcagatgatgtgttattgg
	11631-F	ccaataacacatcatctgaaccccacaATGTCCAAAACGTTCACTAAAAAGTTTGACC
	11631-R	cctgccttaaaggatagaaagactttgttgatGAGAGGGTTACCTCCGGACAAGT
	Tact-11631-F	ACTTGTCCGGAGGTAACCCTCTCatcaacaaagtctttctatcctttaaggcagg
	Padh4-4011-R	TGCTGGTTTGCCGGAGACATtgtggggttcagatgatgtgttattgg
	4011-F	ccaataacacatcatctgaaccccacaATGTCTCCGGCAAACCAGCA
	4011-R	cctgccttaaaggatagaaagactttgttgattAATTGGGCCTCGATCAACTCTAAGTATCG
	Tact-4011-F	CGATACTTAGAGTTGATCGAGGCCCAATTaatcaacaaagtctttctatcctttaaggcagg
	Padh4-1059-R	CCATTTCGCTGAGCTTTACTTAAAGTCATtgtggggttcagatgatgtgttattgg
	1059-F	ccaataacacatcatctgaaccccacaATGACTTTAAGTAAAGCTCAGCGAAATGG
	1059-R	cctgccttaaaggatagaaagactttgttgatCCAGCCAAAGCTCTCGACATG
	Tact-1059-F	CATGTCGAGAGCTTTGGCTGGatcaacaaagtctttctatcctttaaggcagg
Primers for dual-DEG	Tact-8101-dual-R	tacacggtcgactttcttttctttaccacctgccggagctgaagc
	Padh4-1059-dual-F	gcttcagctccggcaggtggtaaagaaaagaaagtcgaccgtgta

Table S3 The relative expression levels of related DEGs in *Phaffia rhodozyma* mutants to the wide type (WT) strain

	M1	M2	M3	M4	M5	M6	M7
Expression level to WT (Fold)	45±3.7	19.4±3.0	5.4±0.5	30.7±4.0	12.7±3.4	1.9±0.4	15.9±2.4/1.6±0.4

M1, mutant of expressing DEG 7463 (alcohol dehydrogenase); M2, mutant of expressing DEG 8101 (NHEJ gene for DNA repair); M3, mutant of expressing DEG 4281 (srf-type TF); M4, mutant of expressing DEG 11631 (aldehyde dehydrogenase); M5, mutant of expressing DEG 4011 (ABC transporter); M6, mutant of expressing DEG 1059 (Ferric reductase); M7, mutant of expressing DEGs 8101 and 1059 (NHEJ gene for DNA repair/Ferric reductase).

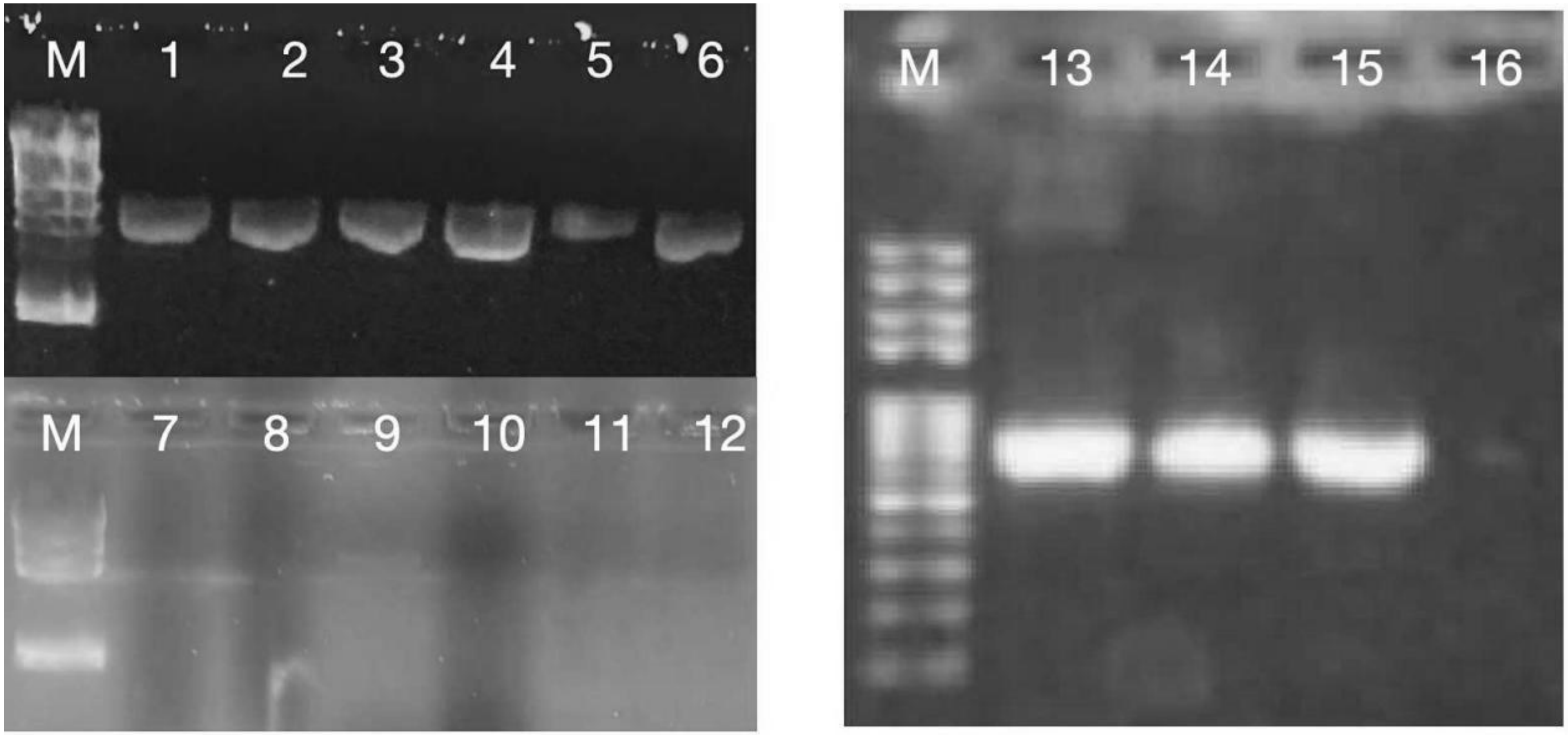

A B

Figure S1 Confirmations of the single- and dual-DEGs expression vectors into genome in *Phaffia rhodozyma* by PCR

A, Single-DEG experssion vectors into genome in *Phaffia rhodozyma*; B, Dual-DEGs expression vector into genome in *Phaffia rhodozyma*

M, DNA markers; line 1-6, integrations of the DEGs' expression vectors, alcohol dehydrogenase, NHEJ gene for DNA repair, srf-type TF, aldehyde dehydrogenase, ABC transporter and ferric reductase, into the genomes of the *P. rhodozyma* mutant 1-6, respectively; line 7-12, the DEGs' expression vectors, alcohol dehydrogenase, NHEJ gene for DNA repair, srf-type TF, aldehyde dehydrogenase, ABC transporter and ferric reductase are not in the genome of the *P. rhodozyma* WT strain; line 13-15, integrations of the dual-DEGs expression vector, NHEJ gene for DNA repair and ferric reductase, in the genomes of the *P. rhodozyma* mutant 7-1, 7-2 and 7-3; line 16, the dual-DEGs' expression vector, NHEJ gene for DNA repair and ferric reductase, is not in the genome of the *P. rhodozyma* WT strain.
